# Ten (not so) simple rules for clinical trial data-sharing

**DOI:** 10.1371/journal.pcbi.1010879

**Published:** 2023-03-09

**Authors:** Claude Pellen, Anne Le Louarn, Gilliosa Spurrier-Bernard, Evelyne Decullier, Jean-Marie Chrétien, Eric Rosenthal, Gérard Le Goff, David Moher, John P. A. Ioannidis, Florian Naudet

**Affiliations:** 1 Univ Rennes, CHU Rennes, Inserm, Irset (Institut de recherche en santé, environnement et travail)—UMR_S 1085, CIC 1414 [(Centre d’Investigation Clinique de Rennes)], Rennes, France; 2 GCS CNCR (Comité National de Coordination de la Recherche), Paris, France; 3 MelanomeFrance, Teilhet, France; 4 Melanoma Patient Network Europe, Uppsala, Sweden; 5 Hospices Civils de Lyon, Pôle Santé Publique, Service REC, Lyon, France; 6 Université de Lyon, Lyon, France; 7 CHU Angers, DRI–Département Science de la Donnée, Angers, France; 8 ANRS|Maladies infectieuses émergentes, PariSanté Campus, Paris, France; 9 France Rein Bretagne, Laillé, France; 10 Centre for Journalology, Clinical Epidemiology Program, Ottawa Hospital Research Institute, Ottawa, Canada; 11 Departments of Medicine, Epidemiology and Population Health, Biomedical Data Science, and Statistics, and Meta-Research Innovation Center at Stanford (METRICS), Stanford University, Stanford, California, United States of America; 12 Institut Universitaire de France (IUF), Paris, France; Carnegie Mellon University, UNITED STATES

## Abstract

Clinical trial data-sharing is seen as an imperative for research integrity and is becoming increasingly encouraged or even required by funders, journals, and other stakeholders. However, early experiences with data-sharing have been disappointing because they are not always conducted properly. Health data is indeed sensitive and not always easy to share in a responsible way. We propose 10 rules for researchers wishing to share their data. These rules cover the majority of elements to be considered in order to start the commendable process of clinical trial data-sharing:

- Rule 1: Abide by local legal and regulatory data protection requirements
- Rule 2: Anticipate the possibility of clinical trial data-sharing before obtaining funding
- Rule 3: Declare your intent to share data in the registration step
- Rule 4: Involve research participants
- Rule 5: Determine the method of data access
- Rule 6: Remember there are several other elements to share
- Rule 7: Do not proceed alone
- Rule 8: Deploy optimal data management to ensure that the data shared is useful
- Rule 9: Minimize risks
- Rule 10: Strive for excellence.

## Introduction

**Fig 1:**
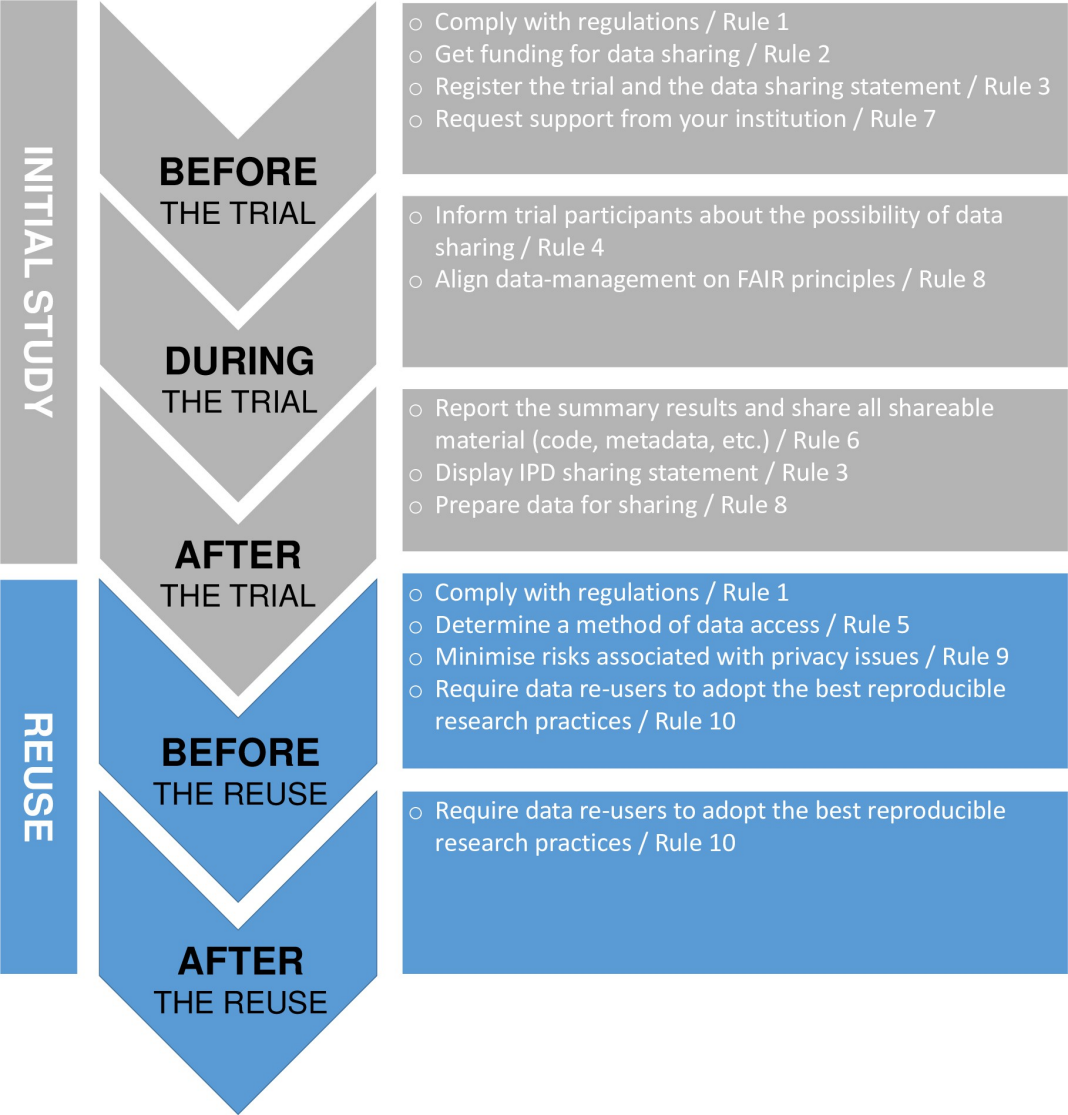
When researchers wishing to share data should implement the 10 rules.

Clinical trial data-sharing is seen as an imperative for research integrity and is becoming increasingly encouraged or even required by funders, journals, and other stakeholders. For example, the White House Office of Science and Technology Policy is now requiring that all data from taxpayer-funded studies be shared without cost or imposition of embargos [1]. Sharing and reusing clinical trial data maximizes its utility [2]. For example, the reanalysis of data from a clinical trial makes it possible to confirm or disprove its results; secondary analyses make way for the exploration of research questions not considered initially, and meta-analyses on individual participant data (IPD) show promise for the field of evidence syntheses. However, early experiences are disappointing with regard to the actual practical implementation of clinical trial data-sharing [3]. Health data is indeed sensitive and not always easy to share in a responsible way. However, responsible reuse of data carried out with the best research standards is a behavior that is not solely desirable but possible provided it is well anticipated. International institutions in some cases provide researchers with comprehensive and complex policies and implementation guidance for sharing data [4]. We propose 10 (not so) simple rules for researchers wishing to share data, aligned on the clinical trial lifecycle (**Fig 1**). These rules cover the majority of elements to be considered in order to start the commendable process of clinical trial data-sharing and to facilitate its efficient use for a new research question.

### Rule 1: Abide by local legal and regulatory data protection requirements

Many legal and regulatory texts govern data-sharing and reuse, especially in terms of data protection. Among the strongest requirements, the European General Data Protection Regulation applies to any organization established in the territory of the European Union or processing data from people residing there. Among other requirements, it imposes the design and maintenance of specific records (e.g., records of processing activities); the provision of information about the reuse is mandatory and the processing of health data is prohibited without a specific exemption, for example for research. In the United States today, the situation is more fragmented and there are both federal and state initiatives and laws that need to be complied with. It is important to familiarize oneself with these requirements, as they also change over time [5].

Since regulatory requirements differ across the world and no harmonization yet exists, it is important to consider the regulatory context of both the data generator and the re-user. For example, a definition of anonymization in the US may not fit in the European context, making the resulting regulatory formalities much more cumbersome. A harmonization of texts to govern clinical trial data-sharing and a simplification of procedures are both certainly needed. This complex regulatory environment reinforces the need for good communication between data generators and data re-users in order to align on the essential information about the requirements in force and the procedures that are to be followed. Thus, researchers should seek support from their institutions and approach data teams if they are identifiable.

### Rule 2: Anticipate the possibility of clinical trial data-sharing before obtaining funding

If one wants to share data, it is important to recall that data-sharing is not resource- or cost-free: data collection, preservation, preparation, and storage in standardized formats, and completion of regulatory and administrative formalities can be time-consuming and resource-intensive processes [6]. A very common barrier to data-sharing cited by trialists is the lack of dedicated financial resources, especially if these costs were not foreseen at the time when the trial obtained funding [7]. It is more and more important to think about data-sharing when the trial is being designed and to request funding dedicated to this activity. This seems feasible, as clinical trial funders are increasingly encouraging researchers to consider how their data will be shared in the future [8]. For example, the National Institute of Health (NIH) has implemented a new data-sharing policy for the research it funds from January 2023 [9]. In France, 5 research-funding agencies (Ademe, Anses, ANR, ANRS|MIE, INCa) have created a working network to harmonize policies regarding open science, and in 2022, they introduced a requirement for a management plan (DMP) [10] for every project approved for funding [11]. Some funders even mandate data-sharing [12]. For example, in Canada, it is the case for the 3 federal funders (Tri-Agency) [13]. Data-sharing is set to become part of grant assessments. For clinical trialists and their respective institutions, to remain competitive, it will be important to adhere to these new requirements. To further educate the research community, and to facilitate data-sharing, the Digital Research Alliance of Canada [14] has recently funded 18 pilot Data Champion programs across the country. In all cases, we recommend that the data flow, including its potential future reuse, should be documented in the DMP in the design stage of the trial. This document, which describes the data collected in a research project, and how it will be structured, shared, and stored, is now mandatory in many institutions. In addition, in the design stages of the clinical trial, researchers should ask their institutions if they have already put in place tools and documents for sharing data. Researchers can draw benefit from existing documents and procedures.

### Rule 3: Declare your intent to share data in the registration step

It is now well established that the key features of clinical trials must be registered before enrolling the first participant [15]. Data-sharing statements are now part of these features: the International Committee of Medical Journal Editors (ICMJE) requires authors to include a statement on data-sharing as part of the clinical trial registration [2]. To be clear, the ICMJE does not enforce data-sharing, so researchers can still answer “no” to this data-sharing plan. Indeed, most data-sharing statements do not lead to prompt, widely available sharing [16] and there are still many obstacles or prerequisites. The statement can be updated on registries to report any change. Importantly, when the trial is over, the data-sharing statement will also be compulsory in the published paper; here again, the data-sharing is not compulsory but the statement is. These data-sharing statements must indicate (i) if individual data will be shared; (ii) which specific data will be shared; (iii) if other documents will be available (including statistical codes); (iv) when the data will be available and for how long; and (v) how access will be provided. Drafting the data-sharing statement is an opportunity to consider how to remove as many obstacles as possible and how to enhance communication with potential re-users in the future. To ensure continuity, named addresses (e.g., corresponding authors) should be avoided, and a valid and permanent email address of the organization that implemented the clinical trial should be provided. The burden of data-sharing should be on the institution, not on the individual researcher; we will return to this later (see Rule 7). A data request form can be developed to facilitate the first exchanges between the organizations of the requester and the data generator and should be accessible for direct download by the re-user, for example, with the annexes of the manuscript or by a link provided in the statement. Here again, researchers should always ask their institutions if such documents have already been developed.

### Rule 4: Involve research participants

A participant-centered approach implies that research projects are carried out “in collaboration with” and not “for” participants. Trial participants should be fully informed of the data-sharing plans prior to entering the trial. When participating in a clinical trial, patients provide their consent to risks and constraints for an uncertain benefit. As data cannot be perfectly anonymized, data-sharing carries a new risk with the possibility of re-identification. Patients must be informed about the use of their data for a different objective from that of the initial clinical trial, about the corresponding risk, and the appropriate safeguards. Informing patients of any possible risks is thus an ethical requirement (to ensure trust, to protect individuals) supporting responsible data-sharing [17] and also a regulatory requirement in some areas, such as Europe. To inform the participants, researchers should provide them with additional information on each specific reuse, check for any potential objection to the reuse and, if there is any, remove the data concerned. Sometimes this objection can target a type of re-user (e.g., industry) or a purpose (e.g., purely commercial). To inform trial participants about reuses, we recommend the use of a website the address of which is provided in advance, for example, in the informed consent form of the trial. Suggested wording for informed consent documents are provided by the Inter-university Consortium for Political and Social Research (ICPSR) [18].

### Rule 5: Determine the method of data access

When data cannot be directly available, the organization that has generated it may have assigned a data access committee or similar body to review the appropriateness of requests for reuse (see Rule 7).

After checking the relevance of the request, the method of data access is usually chosen by the institution on the basis of the security and cost-effectiveness of implementation. For example, to best secure data-sharing, remote access to an infrastructure that incorporates a login option should be used. Remote access to the data also enables the user’s rights to be restricted (e.g., copy and paste data). To achieve this high level of security, it is often necessary to use an external service provider that specializes in providing remote servers. In this spirit, many repositories have been implemented in the USA, such as Vivli and the Yale Open Data Access (YODA) program [19] that already have their own requirements to control and secure data-sharing. Most data from trials that is shareable to date is stored by the industry in these repositories and the rules are already fixed. Researchers can ask their institutions which repository is recommended or use the TRUST principles to ensure that they are reliable [20]. Sometimes it is possible to download the data directly locally onto the re-user’s server; this method has the disadvantage of lowering the level of traceability of the actions carried out on the dataset after sharing. Some tools such as Datashield [21] propose the running of analyses remotely without physically accessing the dataset and only seeing the results (this is called querying the data). Free data download, as proposed by some repositories like Dryad (3), or direct online access avoid the data request steps, but this should be reserved for anonymized data for security reasons. This approach can still be implemented with certain contractual clauses that should be accepted at the time of the download to make the re-user accountable.

### Rule 6: Remember there are several other elements to share

The importance of sharing clinical trial data should not overshadow the basic requirement of complete and transparent reporting of clinical trial results [22]. This includes prospective registration and full reporting of summary results, 2 basic steps that should precede IPD sharing. Prospective registration of the initial study is an essential prerequisite and it enables the main elements of the protocol and its main modifications to be traceable. The reporting of the summary results of the initial study then enables the main results to be communicated in the form of aggregated data. Statistical codes can also easily be shared with fewer restrictions because they do not contain individual data, but they are not so easy to understand and reuse; the name and version of the software and libraries used should be specified along with all other details to make the research reproducible [23]. The most important documents that should be shared are listed in **Table 1**.

**Table 1:** The most important documents to be shared.

*Documents without individual participant data*	*Documents with potential individual participant data*	
	*Anonymization possible (aggregated data)*	*Pseudonymization possible*
- Protocol and its amendments - Statistical analysis plan and its amendments - Statistical codes - Inform consent form template	- Study report - Statistical report - Dataset (in a few cases)	- Dataset (in most cases)

With the possibility of verifying the results, performing secondary analyses, or combining studies in the form of IPD meta-analyses, the correct sharing of data then becomes the icing on the cake.

### Rule 7: Do not proceed alone

Researchers should contact their institutions and seek support from them as soon as they are planning to share data. Indeed, all sponsors, academic or industry, need to set up a governance system for data-sharing and adopt suitable DMPs. To ensure the follow-up of requests, organizations should identify a data reuse coordinator (i.e., the person behind the non-nominative email address described in Rule 3). As a local expert, the data reuse coordinator should be able to guide the re-user in complying with local regulations when necessary. In addition, organizations should set up an independent committee to evaluate requests for reuse of data. This committee will independently evaluate and accept requests for data reuse, as required, on the basis of their scientific and ethical relevance as well as the regulatory, contractual, and financial feasibility. At the very least, to avoid the risk of competing interests, researchers related to the requested study should be excluded from the deliberations of these committees. Lastly, in line with the Hong Kong Principles [24], institutions are invited to implement incentives that reward practices that promote reproducible science, e.g., data-sharing. Many universities, such as the University of Cambridge [25], have therefore developed “data champions programs” that acknowledge the value of good data management. This observation brings us directly to Rule 8.

### Rule 8: Deploy optimal data management to ensure that the data shared is useful

Preparing data for sharing is an essential step that should not be neglected. It requires technical skills and knowledge of regulations to ensure that the data corresponds to the requests of the re-users while respecting regulations. Data management should always aim for compliance with the FAIR principles [26], which ensure that data is Findable [27], Accessible, Interoperable, and Reusable. Similarly, for clinical trial data that includes indigenous participants, the CARE principles [28] should be consulted. The database should use a universal computerized format to enable new research teams to use it easily. Data and metadata should be standardized and preferably in English language. In other words, clinical trial data cannot be kept in an ivory tower nor in the Tower of Babel [29]. Metadata should always be published to enable re-users to easily identify studies of interest. The statistical codes should be annotated to make them understandable by any another statistician [23]. To help the citation of reused datasets, we advise data generators to obtain a DOI name (digital object identifier) for each dataset. Often the entire dataset does not need to be shared. In this case, researchers should create and share a dataset containing only the data needed for reuse. They should also ask re-users to comply with the data citation principles [30] and require them to cite the relevant DOIs in all publications of that reused data.

### Rule 9: Minimize risks

Data-sharing is a risky endeavor from a data protection perspective, because of the risk of re-identification. However, safeguards exist to mitigate this. If the data is publicly available, it should be anonymized. However, anonymizing data does not only raise the issue of potential loss of information from the dataset. Researchers should also expect to undertake a large-scale task, since the rules ensuring the anonymization of data are numerous and not necessarily concordant between countries. For example, one will not obtain the same set of data by following the Health Insurance Portability and Accountability Act (HIPAA) [31] in the US or the data protection working group recommendations [32] in Europe. When anonymization is not possible, researchers can use pseudonymized data, i.e., datasets with no directly identifying data. As re-identification of individuals is possible from these datasets, it is necessary to ensure that the recipients are specified by the protocol and that they are not able to perform analyses that were not agreed on beforehand. Current measures to secure the process often involve (i) establishing a data-sharing agreement that defines the data use rights and obligations; (ii) minimizing the data transmitted, i.e., giving access only to the variables that are strictly necessary for the data reuse; and (iii) choosing the method of data access wisely. However, these security measures sometimes complicate sharing and can therefore appear restrictive but are a guarantee of confidence.

### Rule 10: Strive for excellence

The very idea behind an ethical obligation for clinical trial data-sharing is that researchers should comply with the participants’ wishes—participants having put themselves at risk during the clinical trial—so as to share their data and favoring the best possible use of it [2]. This means that researchers should require data re-users to adopt the highest standards, especially regarding reproducible research practices [33] such as prospective registration of data reuses and the publication of results according to reporting guidelines. It is essential to be as stringent for re-use protocols as for the initial clinical trials. This is of major importance because data reuses can often carry a higher risk of false-positive findings in new analyses than in the initial analysis of the trial which, in theory, have strictly controlled for type 1 and type 2 errors. These exploratory conditions, combined with potential researcher biases, are fertile ground for non-reproducible findings in secondary data analysis, especially when appropriate safeguards, i.e., open science practices, are not in place [34]. Data-sharing efforts should not enable nontransparent selective reporting of new results. In addition, reuse of data can be challenging and often requires good communication between the re-users and the data generators [7]. Ultimately, at the end of this long journey, researchers may well be happy to have shared their data. There are now incentives for the best re-users (e.g., the Parasite Award [35]) and also for data generators (e.g., the Research Symbiont Awards [36]). After all, science should celebrate the dedication of researchers in following these 10 not-so-simple rules.

## Conclusion

**Fig 2:**
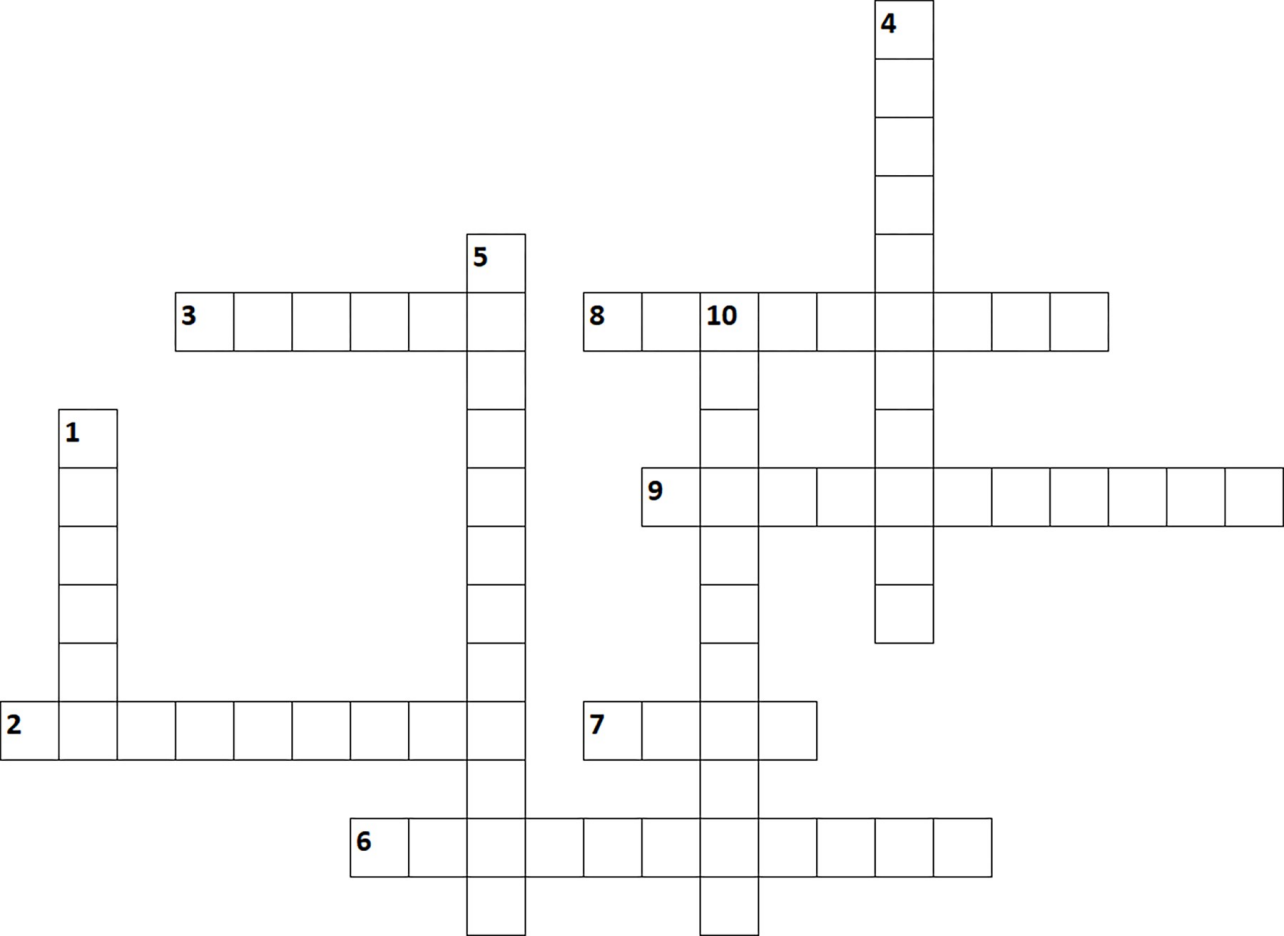
Data-sharing rules crossword.

These 10 simple rules provide various features that will facilitate clinical trial data-sharing. They are not so simple, and in fact rather complex, but **Fig 2** attempts to summarize this complexity in a useable way, so if you don’t get it yet, at least have fun with this:

1. We are all after it and research is often not possible without it.
2. Everyone talks about it but especially the ICMJE, researchers have to publish it in a clinical trials registry and in their articles. This is the data-sharing…
3. Characteristics of the secure server enabling access to the data.
4. Prerequisite for involving participants and ensuring that they do not object to reuse.
5. Step to communicate key elements from the original study protocol prospectively; also recommended for communicating the reuse protocol.
6. Each organization implementing clinical studies should identify this person to manage reuse requests.
7. Principles for ensuring that data is Findable, Accessible, Interoperable, and Reusable.
8. A document that defines the rights and obligations that the data generator and data re-user agree to in any data reuse project.
9. Enables the results of data reuse to be communicated and thus complies with the principles of reproducible research.
10. Unfortunately, they are not universal, but we will have to comply with them for each reuse.

*Answers: 1. Budget; 2. Statement; 3. Remote; 4. Information; 5. Registration; 6. Coordinator; 7. FAIR; 8. Agreement; 9. Publication; 10. Regulations*.

## References

[1] OSTP Issues Guidance to Make Federally Funded Research Freely Available Without Delay | OSTP. In: The White House [Internet]. [cited 2022 Nov 19]. Available from: https://www.whitehouse.gov/ostp/news-updates/2022/08/25/ostp-issues-guidance-to-make-federally-funded-research-freely-available-without-delay/.

[2] Taichman DB, Backus J, Baethge C, Bauchner H, de Leeuw PW, Drazen JM, et al. Sharing Clinical Trial Data: A Proposal from the International Committee of Medical Journal Editors. PLoS Med. 2016;13:e1001950. doi: 10.1371/journal.pmed.1001950 26789528 PMC4720169

[3] Naudet F, Siebert M, Pellen C, Gaba J, Axfors C, Cristea I, et al. Medical journal requirements for clinical trial data sharing: Ripe for improvement. PLoS Med. 2021;18:e1003844. doi: 10.1371/journal.pmed.1003844 34695113 PMC8575305

[4] World Health Organization. Sharing and reuse of health-related data for research purposes: WHO policy and implementation guidance. Geneva: World Health Organization; 2022. Available from: https://apps.who.int/iris/handle/10665/352859.

[5] Group GL. International Comparative Legal Guides. In: International Comparative Legal Guides International Business Reports [Internet]. Global Legal Group; [cited 2022 Jul 4]. Available from: https://iclg.com/practice-areas/data-protection-laws-and-regulations/usa.

[6] Wilhelm EE, Oster E, Shoulson I. Approaches and Costs for Sharing Clinical Research Data. JAMA. 2014;311:1201–1202. doi: 10.1001/jama.2014.850 24556937

[7] Naudet F, Sakarovitch C, Janiaud P, Cristea I, Fanelli D, Moher D, et al. Data sharing and reanalysis of randomized controlled trials in leading biomedical journals with a full data sharing policy: survey of studies published in The BMJ and PLOS Medicine. BMJ. 2018;360:k400. doi: 10.1136/bmj.k400 29440066 PMC5809812

[8] Kiley R, Peatfield T, Hansen J, Reddington F. Data Sharing from Clinical Trials—A Research Funder’s Perspective. N Engl J Med. 2017;377:1990–1992. doi: 10.1056/NEJMsb1708278 29141170

[9] NOT-OD-21-013: Final NIH Policy for Data Management and Sharing. [cited 2022 Nov 7]. Available from: https://grants.nih.gov/grants/guide/notice-files/NOT-OD-21-013.html.

[10] Michener WK. Ten Simple Rules for Creating a Good Data Management Plan. PLoS Comput Biol. 2015;11:e1004525. doi: 10.1371/journal.pcbi.1004525 26492633 PMC4619636

[11] Pernin H, Ancion Z, Guitton S, Rosenthal E, Estaquio C. Comment les agences de financement françaises traduisent-elles leur engagement en faveur de la science ouverte? Partage autour d’un modèle s’appuyant sur une approche concertée. Colloque international Science ouverte au Sud; Cotonou–Bénin; 2022.

[12] Gaba JF, Siebert M, Dupuy A, Moher D, Naudet F. Funders’ data-sharing policies in therapeutic research: A survey of commercial and non-commercial funders. PLoS ONE. 2020;15:e0237464. doi: 10.1371/journal.pone.0237464 32817724 PMC7446799

[13] Government of Canada I. Tri-Agency Research Data Management Policy—Science.gc.ca. Innovation, Science and Economic Development Canada; [cited 2022 Jul 19]. Available from: https://www.science.gc.ca/eic/site/063.nsf/eng/h_97610.html.

[14] Announcing the 2022–2023 Data Champions. In: Digital Research Alliance of Canada [Internet]. [cited 2022 Jul 19]. Available from: https://alliancecan.ca/en/latest/news/announcing-2022-2023-data-champions.

[15] De Angelis C, Drazen JM, Frizelle FA, Haug C, Hoey J, Horton R, et al. Clinical Trial Registration: A Statement from the International Committee of Medical Journal Editors. N Engl J Med. 2004;351:1250–1251. doi: 10.1056/NEJMe048225 15356289

[16] Danchev V, Min Y, Borghi J, Baiocchi M, Ioannidis JPA. Evaluation of Data Sharing After Implementation of the International Committee of Medical Journal Editors Data Sharing Statement Requirement. JAMA Netw Open. 2021;4:e2033972. doi: 10.1001/jamanetworkopen.2020.33972 33507256 PMC7844597

[17] Mello MM, Lieou V, Goodman SN. Clinical Trial Participants’ Views of the Risks and Benefits of Data Sharing. N Engl J Med. 2018;378:2202–2211. doi: 10.1056/NEJMsa1713258 29874542 PMC6057615

[18] Recommended Informed Consent Language for Data Sharing. [cited 2022 Nov 8]. Available from: https://www.icpsr.umich.edu/web/pages/datamanagement/confidentiality/conf-language.html.

[19] The YODA Project | Welcome to the YODA Project. [cited 2022 Jun 16]. Available from: https://yoda.yale.edu/welcome-yoda-project.

[20] Lin D, Crabtree J, Dillo I, Downs RR, Edmunds R, Giaretta D, et al. The TRUST Principles for digital repositories. Sci Data. 2020;7:144. doi: 10.1038/s41597-020-0486-7 32409645 PMC7224370

[21] What is DataSHIELD? In: DataSHIELD [Internet]. [cited 2022 Jun 16]. Available from: https://www.datashield.org/about/about-datashield-collated.

[22] Zarin DA, Tse T. Sharing Individual Participant Data (IPD) within the Context of the Trial Reporting System (TRS). PLoS Med. 2016;13:e1001946. doi: 10.1371/journal.pmed.1001946 26784335 PMC4718525

[23] Sandve GK, Nekrutenko A, Taylor J, Hovig E. Ten Simple Rules for Reproducible Computational Research. PLoS Comput Biol. 2013;9:e1003285. doi: 10.1371/journal.pcbi.1003285 24204232 PMC3812051

[24] Moher D, Bouter L, Kleinert S, Glasziou P, Sham MH, Barbour V, et al. The Hong Kong Principles for assessing researchers: Fostering research integrity. PLoS Biol. 2020;18:e3000737. doi: 10.1371/journal.pbio.3000737 32673304 PMC7365391

[25] Higman R. Data Champions. 15 Nov 2016 [cited 2022 Jun 15]. Available from: https://www.data.cam.ac.uk/intro-data-champions.

[26] Wilkinson MD, Dumontier M, Aalbersberg IJ, Appleton G, Axton M, Baak A, et al. The FAIR Guiding Principles for scientific data management and stewardship. Sci Data. 2016;3:160018. doi: 10.1038/sdata.2016.18 26978244 PMC4792175

[27] Lin S, Ali I, Wilson G. Ten quick tips for making things findable. PLoS Comput Biol. 2020;16:e1008469. doi: 10.1371/journal.pcbi.1008469 33382681 PMC7774837

[28] Carroll SR, Herczog E, Hudson M, Russell K, Stall S. Operationalizing the CARE and FAIR Principles for Indigenous data futures. Sci Data. 2021;8:108. doi: 10.1038/s41597-021-00892-0 33863927 PMC8052430

[29] Grégoire G, Derderian F, Le Lorier J. Selecting the language of the publications included in a meta-analysis: Is there a tower of babel bias? J Clin Epidemiol. 1995;48:159–163. doi: 10.1016/0895-4356(94)00098-b 7853041

[30] Data Citation Synthesis Group. Joint Declaration of Data Citation Principles. Force11. 2014. doi: 10.25490/A97F-EGYK

[31] Rights (OCR) O for C. Summary of the HIPAA Privacy Rule. In: HHS.gov [Internet]. 7 May 2008 [cited 2022 Nov 8]. Available from: https://www.hhs.gov/hipaa/for-professionals/privacy/laws-regulations/index.html.

[32] Article 29 Data Protection Working Party. Opinion 05/2014 on Anonymisation Techniques.

[33] Munafò MR, Nosek BA, Bishop DVM, Button KS, Chambers CD, Percie du Sert N, et al. A manifesto for reproducible science. Nat Hum Behav. 2017;1:1–9. doi: 10.1038/s41562-016-0021 33954258 PMC7610724

[34] Baldwin JR, Pingault J-B, Schoeler T, Sallis HM, Munafò MR. Protecting against researcher bias in secondary data analysis: challenges and potential solutions. Eur J Epidemiol. 2022;37:1–10. doi: 10.1007/s10654-021-00839-0 35025022 PMC8791887

[35] Parasite awards. [cited 2022 Nov 4]. Available from: https://researchparasite.com/.

[36] Research Symbiont Awards. [cited 2022 Nov 4]. Available from: https://researchsymbionts.org/.

[37] Ouvrir la Science—Clinical trial data sharing statement. [cited 2022 Jul 19]. Available from: https://www.ouvrirlascience.fr/clinical-trial-data-sharing-plan/.

